# Synaptic proteins as multi-sensor devices of neurotransmission

**DOI:** 10.1186/1471-2202-7-S1-S4

**Published:** 2006-10-30

**Authors:** Guy Brachya, Chava Yanay, Michal Linial

**Affiliations:** 1 Dept of Biological Chemistry, The Hebrew University of Jerusalem, 91904, Israel

## Abstract

Neuronal communication is tightly regulated in time and space. Following neuronal activation, an electrical signal triggers neurotransmitter (NT) release at the active zone. The process starts by the signal reaching the synapse followed by a fusion of the synaptic vesicle (SV) and diffusion of the released NT in the synaptic cleft. The NT then binds to the appropriate receptor and induces a membrane potential change at the target cell membrane. The entire process is controlled by a fairly small set of synaptic proteins, collectively called SYCONs. The biochemical features of SYCONs underlie the properties of NT release.

SYCONs are characterized by their ability to detect and respond to changes in environmental signals. For example, consider synaptotagmin I (Syt1), a prototype of a protein family with over 20 gene and variants in mammals. Syt1 is a specific example of a multi-sensor device with a large repertoire of discrete states. Several of these states are stimulated by a local concentration of signaling molecules such as Ca2+. The ability of this protein to sense signaling molecules and to adopt multiple biochemical states is shared by other SYCONs such as the synapsins (Syns). Specific biochemical states of Syns determine the accessibility of SV for NT release. Each of these states is defined by a specific alternative spliced variant with a unique profile of phosphorylation modified sites.

The plasticity of the synapse is a direct reflection of SYCON's multiple biochemical states. State transitions occurs in a wide range of time scales, and therefore these molecules need to cope with events that last milliseconds (i.e., exocytosis in fast responding synapses) and with events that can carry on for many minutes (i.e., organization of SV pools). We suggest that SYCONs are optimized throughout evolution as multi-sensor devices. A full repertoire of the switches leading to alternation of protein states and a detailed characterization of protein-protein network within the synapse is critical for the development of a dynamic model of synaptic transmission.

## Background

The structure and function of the nerve terminal has been a topic of extensive research for many years [1,2]. It is only in recent years, however, that the molecular complexity of this structure has been fully appreciated. The availability of complete genomes for different organisms and the development of single molecule detection techniques provided new clues relating to the links between the molecules involved in the structural organization of the synapse and its function [3,4].

### From neuronal properties to molecular properties

Most research in computational neuroscience is conducted from a 'systems biology' perspective. In this approach, each single neuron is considered as an integrator device, joined to a neuronal network [5,6]. A desirable goal is to develop a simulator that will accurately predict the behavior of the neuronal network, and its properties (i.e., synchronization, rhythm, robustness). To accomplish such an ambitious task, it is crucial to incorporate the most accurate biochemical and biophysical properties of all components. There is no single component responsible for the overall neuronal properties. On the contrary, the function of the neuron is a reflection of time and space related events that are sensitive to ion compositions, metabolites and their fluxes, energy supply, second messenger levels, cell anatomy, lipid composition, degree of network connectivity and more. Fortunately, accurate techniques have been developed over the years for direct measurements of neurons in-vivo and in-vitro with high spatial and temporal resolution [7]. Furthermore, as a result of the sequencing of the human genomes and of hundreds of additional genomes, many genomic databases have become available [8,9]. Hence, there is now an almost complete list of genes and proteins that compose the synapse.

We refer to the synapse as the structure of the nerve terminal that is composed of the presynaptic site, the synaptic cleft and the postsynaptic site. Each synapse may have a somewhat specialized composition of proteins and unique architectural properties. Still, it is safe to assume that a generic mammalian synapse will have a set of over 100 different proteins that are distributed between the pre- and postsynaptic sites [10-12]. We limit our discussion to a generic presynaptic site of the mammalian CNS nerve terminal. We will consider a minimal set of ~50 proteins that is directly involved in the maintenance of the presynaptic site and in the exocytosis and endocytosis of SVs [13].

### The "Part List" of pre-synaptic proteins

The components of mammalian presynaptic sites were identified through the use of a combination of genetic and biochemical methods. Most of these proteins belong to gene families and are often also expressed as alternative spliced variants. These proteins are listed in Table 1[11,14].

A static table is unable to capture the dynamic nature of the synapse. Manipulating selected gene products and measuring the biochemical and physiological outcome provided the essential information for reconstructing a dynamic model of the synapse. Such studies lead to a wider view of synaptic proteins. Specifically, replacing the notion of a protein as a single entity with a more elaborate description where each protein sequence is a collection of identified biochemical states. A biochemical state is a static description of a protein beyond its amino acid sequence. This includes a detailed description on all amino acids that are modified and the nature of the modification, the cellular location, the position of small ions and molecules that are bound to the protein, the interaction with other proteins/lipids etc.

What are the 'molecular knobs' that dictate the transition from one biochemical state of a protein to another? How many biochemical defined states are valid in vivo? Are all possible states compatible or alternatively, which ones are mutually exclusive? Can we incorporate the formulation of biochemical protein states for a better understanding of neurological and mental diseases? In this survey, we will illustrate the concept of sequential molecular states in synaptic proteins by exemplifying it for a few key proteins that were postulated in the control of neurotransmitter (NT) release in the synapse.

The effect of switching a protein from one biochemical state to another is a basic tenet of most signal transduction cascades. A switch between alternative states often leads to (i) a change in the protein sub-cellular localization or (ii) a change in affinity towards potential partners, resulting in an alternative protein-protein network. We will not discuss long-term effect in neuronal activity that is often associated with induction of gene expression and with subsequent morphological changes.

### Synaptic vesicle proteins

Many parallel processes take place in the presynaptic site [15]. For example, the organization of the sub-membrane cytomatrix, sorting and trafficking [16], maintenance of membrane potential, establishment of feedback loops for controlling the level of secretion [17] and the partition of SVs into functionally diverse pools [18]. Still, the function of the synapse is by large the regulation of the SV exo- and endocytosis cycle.

Most components that participate in the SV life cycle had been identified a decade ago [19]. However, genetic manipulation of synaptic proteins in model organisms has shed light on their function in the SV life cycle [20]. Probes designed specifically to detect transient protein interacting states were introduced. Among these probes are bacterial neurotoxins [21], fluorescence in vivo dyes that capture a single SV fusion event [22] and antibodies that specifically detect a transient step in the life cycle of SV. Using such probes, the dynamic protein-protein interaction network in the synapse has been characterized [19,23]. It was concluded that only a few tens of proteins participate in the exocytosis and endocytosis cycle in mammalian CNS synapse. We assigned each protein to one of the following functions (i) structure and organisation of the synapse (ii) exocytosis and (iii) endocytosis (Table 1). The actual complexity is much higher as many of the listed proteins belong to relatively large gene families. An extreme example is the Rab family with ~60 genes and multiple variants in mammals [24]. While the list of proteins in the presynaptic site is fairly large, only a few dictate the characteristics of NT release.

Once we focus on proteins directly involved in the SV fusion event, we are left with few proteins of the exocytotic core (yet each may belong to a family of closely related proteins): the SNAREs (VAMP, syntaxin and SNAP-25), their immediate associated protein synaptotagmin (Syt) and the voltage dependent Ca2+ channel (VDCC) [11].

Results from an in vitro reconstitution assay for vesicle fusion showed that the SNAREs in isolation fail to mimic the temporal and spatial characteristics of SV release [25]. However, proteins of the minimal exocytotic core are beautifully designed for the sequence of events leading to SV fusion.

### Proteins as multi-sensor proteins of NT release

The notion that proteins in the synapse act as multi-sensor devices is very attractive as it provides a mechanistic model that relies on the proteins themselves being integrating devices. The requirement for the synapse to release its NT in a fully controllable manner depends on: (i) an efficient coupling between the stimulating signals and the SV life cycle, (ii) an inherent mechanism for sensing the exact physiological state of the synapse, and (iii) a directionality in the molecular intermediates that leads to cycling of exo- and endocytosis. We will elaborate only on the latter aspect.

**Figure 1:**
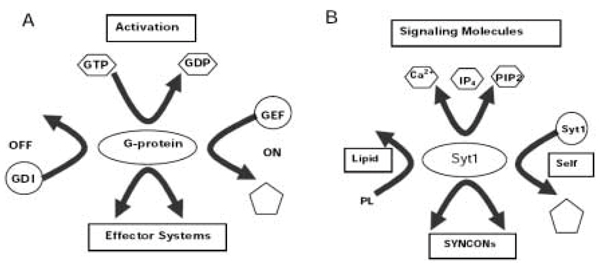
The cycle of activation and inactivation of proteins that drive a cellular process (A) the associated regulators of heterotrimeric G-proteins (B) the cycle of synaptotagmin (Syt) according to interaction with signalling molecules, phospholipids (PL), additional SYNCONs and its self oligomerization (Self). For details see text.

Directionality in an enzymatic reaction is achieved by a coordinated reduction in the substrates associated with an increase in the reaction products. The mechanism that ensures directionality in cellular processes is often dependent on a sequence of conformational changes in some key proteins. A 'textbook' example is the cycle of G-protein activation, one of the best studied switches in signal transduction pathways. G-protein carries an intrinsic GTPase activity that results in a conformational change in the protein, thus switching it from an active to an inactive state (Fig. 1A). The energy cost, in the form of GTP, is associated with each cycle, thus the extent of the reaction reflects the physiological state of the cell. Furthermore, a set of proteins can detect the G-protein in only one out of several alternative conformations which leads to the cyclic operation of the G-protein. The balance among all regulators of G-proteins defines the speed, direction and extent of the signal mediated by the G-proteins.

The G-protein is an example for a protein that is a focal point for regulatory proteins replenishing the cycle. This example is valid also to proteins that control the endo- and exocytosis in nerve terminals (Fig. 1B). A small set of proteins from the presynaptic site, collectively referred to as SYNCONs (Synaptic Control of Neurotransmitter release proteins) are characterized by the following features:

• Binding directly to the exocytotic core

• Having modular structural and functional domain architectures

• Having measurable distinct biochemical states that are dependent on signaling molecules

• The biochemical states are temporary and reversible

• Participating in a transient protein interaction network

• Belonging to protein families that are conserved throughout evolution.

The family of synaptotagmins and specifically synaptotagmin I (Syt1) fully fulfill the features of SYNCONs, thus functioning as multi-sensor devices that dictate the properties of the NT release in most synapses.

#### (i) A genuine protein of the exocytotic core

Synaptotagmin 1 (Syt1) was initially identified as a protein interacting directly with syntaxin [26]. It was identified again in the search for proteins that interact with the α-latrotoxin, the black widow spider neurotoxin that causes a collapse of the control of NT release [27]. Furthermore, Syt1 was independently identified as a partner of the VDCC that is activated in response to action potential in fast synapses [14]. Through this interaction with the VDCC [28] a modulation and competition between Syt1 and the plasma membrane SNAREs (syntaxin and SNAP-25) is confirmed [29]. Thus, Syt1 is a direct modulator of the core proteins of the exocytotic machinery. An additional role of Syt1 in the endocytotic phase of the SV cycle was also confirmed through its direct interaction to AP2 and other components of the endocytotic apparatus (Table 1). Taken together, Syt1 is clearly a genuine part of the core of proteins that control NT release and SV fusion [30].

#### (ii) Modularity in domain architecture

**Figure 2:**
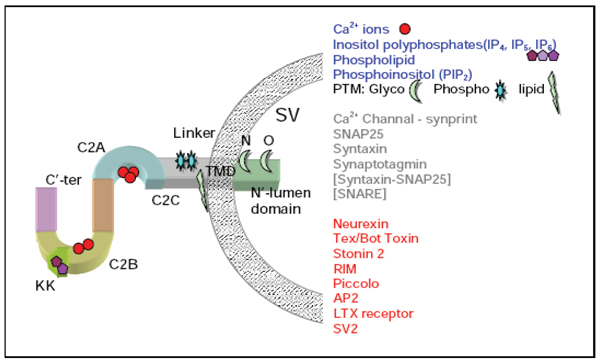
**A schematic view of synaptotagmin1 (Syt1).**  Each functional domain is differently colored. Small molecules that interact with Syt1 (blue letters) and the post translational modifications (black letters) are listed. Proteins of the exocytotic apparatus and their unique combinations are marked in gray. Additional proteins that were shown to interact with Syt1 are shown in red. C2C is the linker sequence between the two C2 domains. Modification sites for N- and O-glycosylations are marked as N- and O-, respectively. TMD, transmembrane domain; KK, a stretch of basic residues that defines the binding site for inositol polyphosphates [30].

Syt1 is composed of the following modular units: a short N-terminal luminal sequence, a single transmembrane (TMD) domain, a linker sequence and two C2 domains (the C2A and C2B domains). The protein is localized to the membrane of the SV with the C2 domains facing the cytoplasm (Fig. 2) [31,32]. This basic modular structure is maintained throughout evolution and is identical to that found in fly, worm, frog, snail, squid and more [33]. Syt1 is composed of few autonomous domains (i.e. C2A, C2B, luminal domain, TMD). Such design allows each domain to act almost independently in sensing different aspects of cell physiology. Such modularity is the basis for the properties of Syt1 as a multi-sensor. Interestingly, several Syt-like proteins expressed in the brain that lack TMD were identified. The role of these molecules as competitors for Syt1 is currently being tested (Linial, unpublished).

#### (iii) Dynamic sensor for small signaling molecules

**Figure 3:**
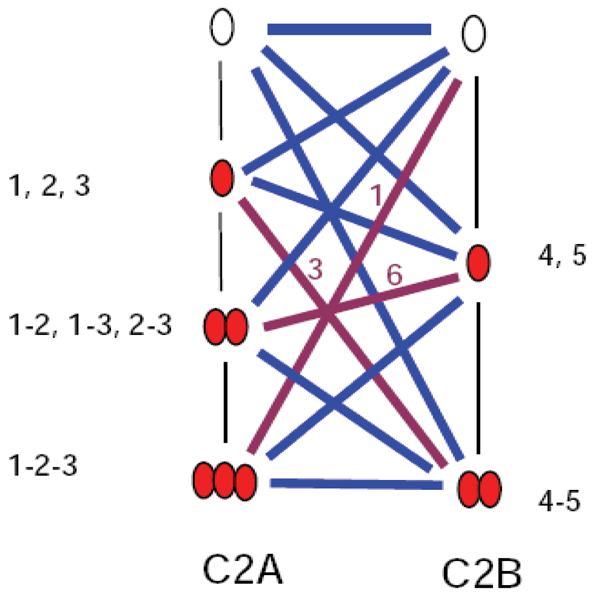
**Schematic illustration of the Ca2+ occupancy states for Syt1.** The maximal number of Ca2+ ions (symbolized as red ovals) that can bind C2A is 3 (marked as 1,2,3) and 2 (marked as 4, 5) for the C2B. The blue and purple lines represent all potential states ranging from no binding to maximal occupancy by 5 ions. Total of 12 edges representing Syt1 states assuming the actual position of the ions within C2A or C2B is not important. Addition of the positional information increases the number of Ca2+ occupancy states as illustrated by the purple edges. The number of individual states associated with the purple edges summarizes to ten combinations of occupancy of 3 Ca2+ ions. With positional information for Ca2+ ions occupancy the number of individual states reaches 32 (1 state for no occupancy, 5 states for one ion, 10 states for 2 ions, 10 states for 3 ions, 5 states for 4 ions and another state for occupancy of 5 ions).

The C2A domain of Syt1 may bind three Ca2+ ions and the C2B domain two Ca2+ ions [33,34]. This biochemical observation is supported by structural studies, as well as by direct mutagenesis of key residues that coordinate the Ca2+ ions [31]. The intrinsic Ca2+ affinity of the C2 domains is very low but following binding to the negatively charged phospholipid membranes, the affinity increases by ~1000 fold to reach a micromolar range. The binding of Ca2+ to C2 domains induces a change in the molecule that can be now viewed as a new (biochemical) state. A link between the number of bound ions in Syt1 and the capacity of the protein to act as a sensor for exocytosis was shown [35]. Considering all possible combination of Ca2+ occupancy one can theoretically list 12 'states' for Syt1 with the simplified assumption that only the number of Ca2+ ions and not their exact position is relevant. This number can reach 32 individual states once the actual position of each of the Ca2+ ions is considered. Illustration of the rich repertoire of potential biochemical states in view of Ca2+ occupancy is shown in Fig. 3.

Syt1 is capable of binding and integrating information for additional signaling molecules such as the inositol tetrakisphosphate (IP4) and other inositol polyphosphates (IP5 and IP6). These molecules bind to the basic amino acid stretch within the C2B domain (Fig. 2). The affinity of Syt1 to Ca2+ is modulated according to the binding of inositol polyphosphate moieties [30], the by-products of extensive stimulation of the nerve terminals. The concentration of free Ca2+ and IP4 in the synapse defines a fairly complex binding competition curve for Syt1. In view of Syt1, the number of biochemical defined states becomes enormous. Syt1 states that are defined by Ca2+ and IP4 reflect the status of the synapse for a time scale that is in the millisecond and tens of milliseconds range, respectively.

#### (iv) Protein-protein interaction network

The signaling molecules that bind to Syt1 are a direct reflection of the level of activity of that synapse. How is the molecule able to transmit and convey this information to the exocytosis machinery and in particular to the fusion pore? The SNARE proteins can be viewed as the minimal set of proteins necessary to facilitate the creation of the fusion pore. Studies that addressed the importance of Syt1 were performed in-vitro in a minimal fusion setting or in a physiologically impaired setting [36]. The vesicle fusion in such artificial systems takes minutes rather than milliseconds. In the absence of Syt1, the SNAREs lack the 'stimulus-response' coupling expected from the exocytotic event in a functional synapse. The affinity of Syt1 to the exocytotic machinery is Ca2+ and IP4 dependent. This is in addition to the intrinsic capacity Syt1 exhibits in isolation for binding these signaling molecules (Fig. 2). Binding Syt1 to SNARE complexes has two components, Ca2+ dependent and independent. Syt1 can bind to the preassembled SNARE complex in the absence of Ca2+, but as Ca2+ flows in, it switches to the phospholipid membrane and inserts some of the hydrophobic residues into the lipid layer (Fig 1B). The assumption is that binding of C2 domains to the phospholipids accelerates the assembly of the SNARE dependent fusion pore. An additional facet of the Syt1 biochemical 'multi state' model is the ability of the molecule to oligomerize (Fig. 1B). Syt1 can form homodimers and even higher orders of multimers via its own TMD or directly via the C2B domain. But, only the C2B dependent oligomerization is Ca2+-dependent. A short conserved region of polybasic residues (Fig 2, KK) in the C2B domain was shown to be critical for the Ca2+-dependent NT release and also for the binding to syntaxin-SNAP25 pair. This pair is a prerequisite for the assembly of the SNARE complex. Considering this, Syt1 may be essential to coordinate the fine apposition of the vesicle, relative to the Ca2+ channel at the initiation of the fusion pore [37]. In the event that IP4 binds to these polybasic residues, it inhibits SNARE interaction competitively [33]. While we have focused on the delicate balance of Syt1-SNAREs interaction in binding of the signaling molecule, other synaptic molecules (i.e. AP2 endocytotic adaptor, Neurexin, SV2, the Ca2+ channels, Fig. 2) are typically sensitive to the Syt1 biochemical status as well.

#### (v) Reversibility of biochemical states

Most of the discussion above relies on in-vitro binding experiments. However, post-translational modification (PTM) of Syt1 provides more information on 'stimulus response' coupling. There are three sites of phosphorylation (for different kinases) [38], two sites of glycosylation at the N' luminal terminal and sites for lipid modification in the vicinity of the TMD. All these modifications were confirmed in vivo and in vitro (Fig. 2). More significantly, the phosphorylation state of the molecule defines the degree of interaction with syntaxin-SNAP25 pair, and therefore may rebalance the chain of reaction leading to Ca2+ dependent SV fusion. These PTMs increase enormously the number of biochemical states of Syt1. These states may drive (or inhibit) the interaction with additional large number of partner proteins. The activity of different kinases and phosphatases in the synapse is often associated with the level of plasticity, learning and memory [39]. It is thus tempting to postulate that SYNCONs are the first to respond to these modifying enzymes.

#### (vi) Protein family – evolutionary perspective

Syts in human belong to a gene family composed of about 17 genes and several related Syt-like proteins [40]. Some of the genes also seem to be expressed as different spliced variants [41]. A distinction in biochemical properties for the different members of the Syt family was drawn in view of (i) their affinity for divalent ions such as Ca2+ and Sr2+; (ii) binding a spectrum of signaling molecules such as IP4 and (iii) binding to lipids such as phosphatidylinositol 4,5-bisphosphate (PIP2), a plasma membrane lipid with an essential role in exocytosis and endocytosis. (iv) post-translational modifications (v) interacting mode with the membrane; (vi) sensitivity of the promoters to stress and hyper excitation and more. For example, Syt 7 is ~400-fold more sensitive to Ca2+ than is Syt1. By replacing Ca2+ by Sr2+ and Ba2+ an uncoupling of the binding to lipid and to t- SNAREs is achieved. Only few of the Syts exhibit such uncoupling [42]. The complexity of mammalian CNS can explain some of this intricacy, with several of the Syt proteins expressed in the same cells. Furthermore, tight regulation of expression of only some members of the Syt family appears to be associated with pathological states.

### Multi-sensor proteins for presynaptic site organization

The time-scale of Syt1 in the control of NT release ranges from millisecond (exocytosis) to seconds (endocytosis). Other presynaptic proteins such as synapsins (Syns) act in a much longer time frame ranging from minutes (facilitation) to days (plasticity). Syns are a set of proteins sharing features characteristic to SYNCONs, serve as multi-sensor devices that modulate the organization of SV and in general the architecture of presynaptic site [43].

In mammals, there are 3 genes that account for multiple alternatively spliced variants of syn that are expressed in the CNS. Knockout mice for one or even two or the Syn genes showed only mild effects on brain development. The main function of Syns is to bridge SVs to the cytoskeleton mesh through an interaction of F-actin and the SV membranes [44]. Consequently, Syns affect the gross morphology of the presynaptic site and primarily the accessibility of SVs to engage in productive cycles of exo-endocytosis.

What are the knobs that allow the switch of Syn from one biochemical state to the other? All 3 Syn genes bind ATP with a high affinity in a shared central domain of the protein. But while Syn1 binds ATP only in the presence of Ca2+, Syn2 binds ATP irrespective of Ca2+, and Syn3 binds ATP only in the absence of Ca2+ [45]. Furthermore, each of the Syn variants acts as a substrate for a combination of kinases and phosphatases [46,47]. Repression and activation of these kinases and phosphatases are a direct consequence of the amounts of signaling molecules such as calmodulin, Ca2+ and cAMP. The outcome is a phosphorylation-dependent alteration in the capacity of Syns to crosslink the SV with the actin mesh [48]. All Syn variants have a phosphorylation site for PKA and for CaMK I/IV that is conserved across vertebrate and invertebrates [49].  Syns can also be phosphorylated by CaMK II [50], mitogen-activated protein kinase Erk, and cyclin dependent kinase [51]. Thus, the actual level of phosphorylation and their exact position on the protein produce a range of biochemical entities that together determine the potential of the synapse to efficiently respond to stimulation. As for the other SYNCONs, Syns are engaged in multiple transient protein-protein interactions (Table 2). Syn was proposed as an effector of Rab3, namely, its binding is fully dependent on Rab3 GTP/GDP state [52]. Rab3 is a small G-protein binding protein that is as a master switch that determines the compatibility of SV for release [53]. The binding of Syt for some partners is not only Ca2+ phosphorylation dependent but directly Ca2+ dependent (i.e., S100A) [54]. In addition to the molecular and cellular level, mutations or polymorphic sites of these genes associated with neurological impairments [55,56]. Human genetic data ties specific polymorphic sites in Syn1 gene with the ethiology and pathology of psychiatric disorders. In a family with members affected by epilepsy, learning disabilities and behavior disorders a mis-sense mutation in Syn1 was detected. Similarly, a mutation in Syn3 resulted in learning impairments. The involvement of different Syn variants in protein interactions and in pathological conditions is summarized in Table 2.

### Synaptic proteins as information integrators

To fully appreciate the potential of molecules such as Syt1 and Syns as 'coincidence detector devices', quantitative parameters on binding properties, on competition and affinities and on modification of the molecules under physiological setting are essential. Although our discussion is limited to only two examples of SYNCONs, additional molecules in the synapse show similar molecular coupling. A common observation for the 'multi-sensor devices' which function in controlling NT transmission is that mutations or even a subtle alteration in expression level cause impairment in learning and behaviour. An example is the NMDA glutamate receptor of the postsynaptic membrane. NMDA controls aspects of plasticity, neurotoxicity, learning and memory and is involved in the symptoms of schizophrenia and other conditions [57]. Like Syt1 and Syns NMDA participates in a dynamic protein-protein interaction network.  The activity of NMDA depends on post-translation modification scheme, on variation in subunit assembly, alternative spliced variants, trafficking, localization, internalization properties and the binding of multiple ligands and ions. Integration of all those variables specifies a large repertoire of NMDA variants that are identified by their biophysical and biochemical properties. While NT release and postsynaptic response are controlled by SYNCONs and NMDA, respectively, additional multi-sensor proteins function in the synapse. It was proposed that domains in a set of endocytotic proteins (i.e., Ampiphysin, Table 1) act as multi-sensor by binding to different microenvironment of lipids. As a result a membrane curvature is induced. Of course, the efficiency of vesicle fusion, fission and cell division is strongly dependent on the appropriate membrane curvature [58].

## Conclusion

The detailed analysis of Syts and Syns is used to illustrate a common theme unifying all SYNCONs. Integration of comparative genomic view, expression profile, biochemical data, subcellular localization and protein-protein interactions is needed to describe the function of SYNCONs as proteins that adopt many discrete states and thus play a fundamental role as multi-detector devices of the synapse. Additional presynaptic proteins like Munc13, Munc18, RIM and Rab3 (Table 1) share many of the features of SYNCONs, though they function in a time scale suitable for SVs maintenance and recovery [59]. A dynamic model of functional synapse will include in-vivo measurements from various SYNCONs. Such a model will be the basis for constructing accurate simulation of a behaving synapse and eventually, of a functional circuit in the brain.

## Abbreviations

NT, neurotransmitter, SYNCON, Synaptic control of neurotransmitter release protein; SNARE, SNAP receptors; SV, synaptic vesicle; Syt, synaptotagmin; Syn, synapsin; TMD, transmembrane domain; VDCC, voltage-dependent Ca2+ channels.

## Authors' contributions

All authors participated in developing the ideas, the writing, discussion and integration of the information. A preliminary version of this paper was presented in SYMBIONIC international school in Trieste 2005.

## Figures and Tables

**Table 1:** A list of major pre-synaptic proteins in the mammalian synapse.

Protein	ID UniProt	Symbol HUGO^a^	Localization	TMD	Comment^b^
1. Amisyn	Q8NFX7	STXBP6	Cyt	No	Exo
2. Amphyphysin	P49418	AMPH	SV, PM	No	Endo
3. Basoon	Q9UPA5	BSN	Cyt	No	M
4. Bridging integrator	O00499	BIN1	Cyt, SV	No	M*
5. CAPS	Q9ULU8	CADPS	Cyt, SV	No	Exo
6. CASK	O14936	CASK	Cyt	No	M. Exo
7. CASKIN	Q8WXD9	CASKIN1	Cyt, PM	No	M. Exo
8. CAST	O15083	CAST1	Cyt, PM	No	M
9. Complexin	Q6PUV4	CPLX2	PM	No	Exo
10. CSP	Q9H3Z4	DNAJC5	Cyt, SV	No	M, Exo
11. Doc2	DOC2A	Q14183	SV	No	Exo
12. Dynamin	P50570	DNM2	Cyt, SV	No	M, Endo
13. Epsin	Q9Y6I3	EPN1	Cyt	No	M, Endo
14. Hrs-2	O14964	HGS	Cyt, SV	No	M
15. Intersectin	Q15811	ITSN1	CytSV	NoNo	Endo, M
16. Latrophilin	O94910	LPHN1	PM	Yes	Exo
17. Liprin-α	Q13136	PPFIA1	Cyt	No	M*
18. Mint/X -11	Q02410	APBA1	Cyt	No	M, Exo
19. Munc-13	Q9UPW8	UNC13A	Cyt, PM	No	M, Exo -Endo
20. Munc-18	O00186	STXBP3	Cyt, PM	No	Exo
21. Neurexin	P58400	NRX1	PM	Yes	M, Exo
22. NSF	NSF	P46459	SV, Cyt	No	M*
23. NT transporter	P23975	SLC6A2	SVPM	Yes	M
24. NTvesicle transporter	Q16572	SLC18A3	PMSV	Yes	M
25. P/Q Ca2+ channel	O00555	CACNA1A	PM	Yes	Exo
26. Pallklin	Q9UL45	PLDN	Cyt, SV	No	M
27. Piccolo	Q9Y6VO	PCLO	Cyt	No	M
28. Rab3	P20336	RAB3A	Cyt, SV	No	M, Exo *
29. Rabconnectin-3	Q8WTV7	DMXL2	Cyt	No	M, Exo
30. Rabphilin	Q9Y2JO	RPH3A	SV	No	M, Exo
31. RIM	Q86UR5	RIMS1	PM	No	M, Exo
32. RIMS binding protein	O15034	RIMBP2	SV	Yes	Exo, M *
33. SCAMP	O15126	SCAMP1	SV	Yes	M
34. SNAP-25	P60880	SNAP25	PM	No	Exo
35. Snapin	O95295	SNAPAP	PM, Cyt	No	Exo, Endo
36. Stonin-2	Q8WXE9	STN2	Cyt	No	Endo
37. SV2	Q7L0J3	SV2A	SV	Yes	Exo, M
38. Synapsin	P17600	SYN1	SV	No	M
39. synaptogyrin	O43759	SYNGR1	SV	Yes	Exo
40. Synaptojanin	O43426	SYNJ1	Cyt PM	No	Endo*
41. Synaptophysin	P08247	SYP	SV	Yes	M, Exo
42. Synaptotagmin	P21579	SYT1	SV	Yes	Exo, Endo
43. Syntaphilin	O15079	SNPH	PM	Yes	Exo
44. Syntaptoporin	Q8TBG9	SYNPR	PM	Yes	Exo
45. Syntaxin	Q16623	STX1A	PM	Yes	Exo
46. Tomosyn	Q5T5C0	STXBP5	PM	Yes	Exo
47. VAMP	P63027	VAMP1	SV	Yes	Exo
48. VAT-1	Q99536	VAT1	SV	No	M
49. Vo	P27449	ATP6V0C	PM	Yes	Exo, Endo
50. α-SNAP	P54920	NAPA	SV	No	M, Exo

Most proteins that function in the mammalian SV life cycle are listed. ^a^Protein symbols are provided by HUGO, the Human Genome Organization Gene nomenclature committee . IDs are according to UniProt database . ^b^Proteins listed are from Human are marked according to their main function in exocytosis (Exo), endocytosis (Endo) and synapse maintenance (M). The proteins with intrinsic enzymatic activity are marked by an asterisk (*). Proteins whose function is in building, shaping and maintenance of the pre-and postsynaptic structures are not included. For simplicity only one representative of a gene family is listed. PM, plasma membrane; Cyt, cytosol; TMD, transmembrane domain.

**Table 2:** Human synapsin (Syn) gene family.

Protein	Length (aa)^a^	Possible Exons	Human-chromosome	Disease	Interactions
Synapsin 1 (Syn1)	705/669/261	16	Xp11.23	ALZ, epilepsy	Rab3, S100A1, Actin, tubulin, CAPON, Syn1, Syn2, Syn3
Synapsin 2 (Syn2)	505/401/301/285	21	3p25.2	schizophrenia	Actin, CAPON, PLCγ1, Syn1, Syn2, Syn3
Synapsin 3 (Syn3)	580/579/456/451/236/192/(+ 4)	31	22q12.3	Schizophrenia Bipolar disorder Multiple sclerosis	Actin, CAPON, Syn1, Syn2, Syn3

^a^Variants listed are alternatively spliced forms. The expression of some of these variants is detected only under extreme stimulation conditions. The expression of some of these variants was not confirmed experimentally. Syns can form homo- and heterocomplexes among their family members.
